# Novel *GPRC5B*::*PRKCA* gene fusion in an unclassifiable low-grade glioneuronal tumor

**DOI:** 10.1093/noajnl/vdag156

**Published:** 2026-06-08

**Authors:** Yasuhide Takeuchi, Yohei Mineharu, Yukinori Terada, Noritaka Sano, Masahiro Tanji, Naoki Goda, Masayuki Hara, Yuki Oichi, Takahiko Kamata, Keita Fukuyama, Yoshihiro Yamamoto, Sachiko Minamiguchi, Manabu Muto, Susumu Miyamoto, Hironori Haga, Yoshiki Arakawa

**Affiliations:** Department of Diagnostic Pathology, Kyoto University Graduate School of Medicine, Kyoto, Japan; Department of Neurosurgery, Kyoto University Graduate School of Medicine, Kyoto, Japan; Department of Neurosurgery, Kyoto University Graduate School of Medicine, Kyoto, Japan; Department of Neurosurgery, Kyoto University Graduate School of Medicine, Kyoto, Japan; Department of Neurosurgery, Kyoto University Graduate School of Medicine, Kyoto, Japan; Department of Diagnostic Pathology, Kyoto University Graduate School of Medicine, Kyoto, Japan; Department of Diagnostic Pathology, Kyoto University Graduate School of Medicine, Kyoto, Japan; Department of Neurosurgery, Kyoto University Graduate School of Medicine, Kyoto, Japan; Department of Neurosurgery, Kyoto University Graduate School of Medicine, Kyoto, Japan; Department of Medical Oncology, Kyoto University Graduate School of Medicine, Kyoto, Japan; Department of Medical Oncology, Kyoto University Graduate School of Medicine, Kyoto, Japan; Department of Diagnostic Pathology, Kyoto University Graduate School of Medicine, Kyoto, Japan; Department of Medical Oncology, Kyoto University Graduate School of Medicine, Kyoto, Japan; Department of Neurosurgery, Kyoto University Graduate School of Medicine, Kyoto, Japan; Department of Diagnostic Pathology, Kyoto University Graduate School of Medicine, Kyoto, Japan; Department of Neurosurgery, Kyoto University Graduate School of Medicine, Kyoto, Japan

A 14-year-old boy presented with an incidentally identified lesion in the right basal ganglia. MRI revealed a well-demarcated lesion with focal enhancement and no significant edema. Subtotal resection was performed, and pathology revealed an unclassifiable mixed glial and neuronal tumor without papillary/pseudopapillary structures and was immunoreactive for OLIG2, synaptophysin, and NeuN. RNA sequencing revealed a novel in-frame *GPRC5B*::*PRKCA* fusion gene. The tumor was diagnosed as a low-grade glioneuronal tumor, not elsewhere classified. This intracranial tumor was the first case of *GPRC5B*::*PRKCA* gene fusion showing characteristic histological findings.

A novel *GPRC5B*::*PRKCA* gene fusion in an unclassifiable cerebral low-grade glioneuronal tumor is reported.

An otherwise healthy 14-year-old boy was referred to our hospital for an incidentally identified nodular lesion in the right basal ganglia seen on CT performed after a traumatic fall. A nodular lesion (approximately 1.5 cm at the largest diameter) with peripheral heterogeneous calcification of the tumor (Figure 1A) was seen in the right basal ganglia. On MRI, the lesion occupied the region between the head of the right caudate nucleus and the ventral side of the right putamen; it was hypointense on T1-weighted images and hyperintense on T2-weighted images (Figure 1B). Focal enhancement of the dorsal component of the tumor was observed. The tumor was well-defined, and there was no peritumoral brain edema on the T2-weighted and fluid-attenuated inversion-recovery imaging sequences (Figure 1C). The preoperative differential diagnosis included low-grade astrocytoma and ganglioglioma. The first biopsy showed scattered microcalcification without proliferative tumor cells. At 4-year follow up, the tumor had gradually enlarged to approximately 2.0 cm at the largest diameter (Figure 1D and E). A second biopsy was performed at the age of 18. The postoperative course was uneventful. Follow-up MRI examinations were performed 7 times over a 6-year period after the second biopsy and showed no tumor growth or neurological deterioration.

**Figure 1.**
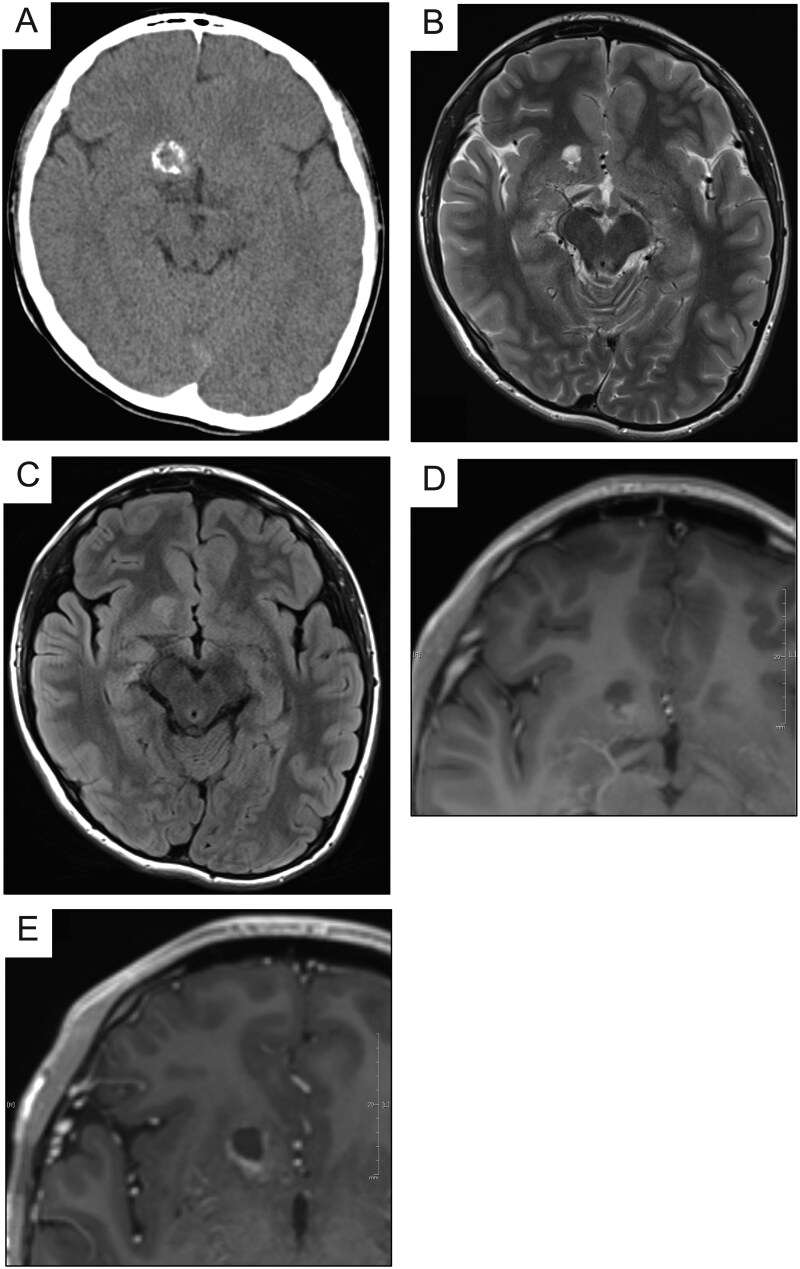
Details of imaging findings. (A) Computed tomography (CT) images at initial diagnosis showing a nodular lesion with heterogeneous calcification in the right basal ganglia. (B) T2-weighted magnetic resonance (MR) image showing a hyperintense lesion between the head of the right caudate nucleus and ventral putamen. (C) MRI fluid-attenuated inversion-recovery imaging (FLAIR) showing no peritumoral edema. (D, E) Gadolinium-enhanced T1-weighted MRI images showing heterogeneous enhancement at diagnosis (D) and tumor enlargement to ∼2.0 cm after 4 years (E).

Histopathological examination of the second biopsy specimen showed heterogeneous features. Cellular areas showed diffuse proliferation of tumor cells with uniform, small round nuclei within a background of delicate neuropil-like matrix (Figure 2A). The chromatin showed a “salt-and-pepper” appearance with occasional perinuclear halos. Mitotic figures, necrosis, and microvascular proliferation were not identified. Papillary/seudopapillary structures and well-formed neurocytic rosettes were not clearly identified, although clusters of tumor cells were present. Less cellular areas contained abundant Rosenthal fibers within a glial fibrillary background, accompanied by scattered tumor cells with irregular hyperchromatic nuclei (Figure 2B). Calcifications were observed in the non-tumorous cerebral tissue (Figure 2C). Because the biopsy specimens were fragmented, infiltration of the surrounding brain tissue could not be reliably assessed. Immunohistochemical analyses showed that tumor cells in the cellular areas were diffusely positive for OLIG2, synaptophysin, and NeuN, and negative for glial fibrillary acidic protein (GFAP) (Figure 2D-F), supporting neuronal differentiation with glial lineage features. In contrast, the Rosenthal fiber–rich areas demonstrated GFAP-positive glial processes with scattered OLIG2-positive cells. Tumor cells were negative for IDH1 (R132H), and CD34. The Ki-67 labeling index was less than 1% (Figure 2G). Fluorescence in situ hybridization showed no 1p/19q codeletion.

**Figure 2.**
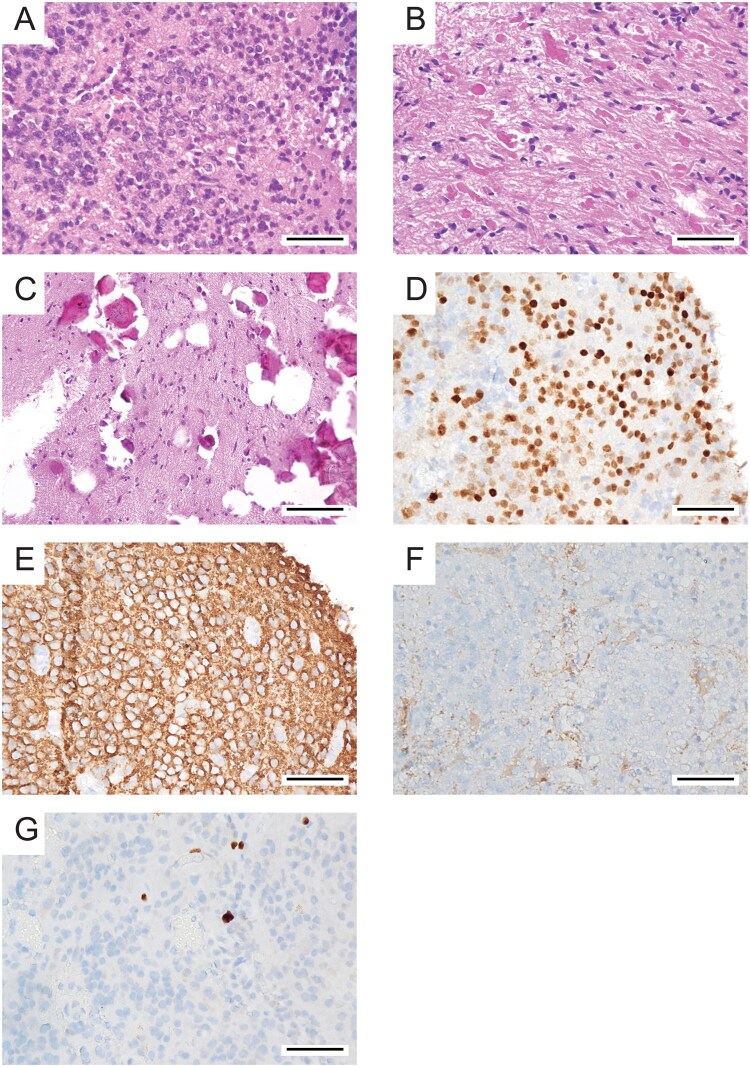
Details of histological findings. (A, B) Microscopic images of the second biopsy specimen hematoxylin and eosin (H&E) staining (400×) showing cellular areas composed of small round tumor cells (A) and less cellular areas with irregular hyperchromatic nuclei and abundant Rosenthal fibers (B). (C) H&E staining of the second biopsy specimen (200×) showing scattered calcifications. (D, E) Tumor cells are positive for OLIG2 (d) and synaptophysin (e) (400×). (F) Tumor cells are negative for GFAP (400×). (G) Ki-67 labeling index is less than 1% (x40). Scale bars: (A, B, D-G) 50 µm; (C) 100 µm.

Because the tumor could not be classified histopathologically, mRNA sequencing was performed on snap-frozen tissue obtained from the second biopsy, as previously described,^1^ which identified *GPRC5B*::*PRKCA* fusion (Figure 3A). The fusion was validated via reverse transcription polymerase chain reaction, confirming an in-frame fusion between the 3-prime end of *GPRC5B* exon 2 and the 5-prime end of *PRKCA* exon 5 (Figure 3B). No fusions involving *FGFR1*, *TACC1*, *TACC3*, or *SLC44A1* were detected. DNA was extracted from frozen tumor tissue obtained from the second biopsy (separate from the batch used for RNA extraction), subjected to methylation profiling using Infinium Human MethylationEPIC BeadChip (Illumina Inc.), and analyzed using the DKFZ methylation classifier^2^ (v12.8). The tissue was classified as a reactive tumor microenvironment (calibrated score of 0.944493), without significant copy number alterations (data not shown). Using NIH classifier, the sample was classified as CONTR REACT class (score 0.995) (https://methylscape.ccr.cancer.gov/. Accessed March 11, 2026). Histopathological evaluation of the formalin-fixed paraffin-embedded (FFPE) tissue estimated the tumor cell content to be approximately 70%, but methylation analysis on FFPE tissue could not be performed due to insufficient residual material. Because the frozen specimen was submitted directly for molecular analysis, the precise tumor cellularity could not be reliably assessed. Detailed molecular assays are described in the Supplementary Text File.

**Figure 3.**
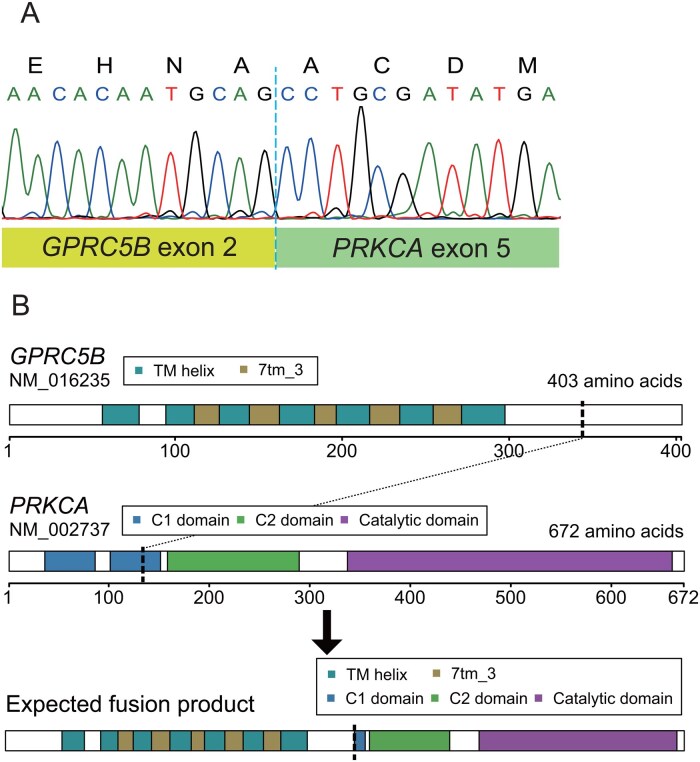
Details of genomic findings. (A) Sanger sequencing confirming an in-frame *GPRC5B*::*PRKCA* fusion. (B) Functional domains of *GPRC5B*, *PRKCA*, and the fusion product expected by the sequencing analysis, together with fusion sites (*GPRC5B* exon 2 and *PRKCA* exon 5). TM: transmembrane, 7tm_3: 7 transmembrane sweet-taste receptor of 3 GPRC

Considering the histological features and molecular findings, the tumor was diagnosed as a low-grade glioneuronal tumor, not elsewhere classified.^3^

In central nervous system tumors, *GPRC5B*::*PRKCA* fusion has not previously been described. *GPRC5B* encodes a class C orphan G protein–coupled receptor with 7 transmembrane domains that is highly expressed in the brain and may play a role in central nervous system homeostasis.^4^  *PRKCA* encodes protein kinase C-α (PKCα), a serine/threonine kinase involved in multiple cellular processes.^5^ In the central nervous system, PRKCA fusions are characteristic of papillary glioneuronal tumor (PGNT), most commonly *SLC44A1*::*PRKCA*, although other partners such as *NOTCH1* and *GPR37L1* have also been reported.^6^

PRKCA contains an N-terminal regulatory domain with C1/C2 motifs and a C-terminal catalytic kinase domain. In the present case, the fusion deletes exons 1-4, resulting in partial loss of the regulatory region including part of the C1 domain. Previous studies suggest that PKC catalytic-domain fusions may be constitutively active but also prone to degradation through cellular quality-control mechanisms.^7^ Most reported PRKCA fusions involve breakpoints at exon 9 or later, whereas the breakpoint in the present case occurs at exon 5 and retains part of the regulatory region.^6^ Therefore, the functional consequences remain uncertain. Loss of regulatory elements may alter intracellular localization of PKC. Collectively, the fusion may dysregulate kinase activity or intracellular localization and thereby contribute to tumorigenesis, although the precise mechanism remains unclear.

PGNT was considered in the differential diagnosis because of the *PRKCA* fusion. However, the tumor lacked characteristic pseudopapillary structures composed of GFAP-positive glial cells and interpapillary neuronal elements, and DNA methylation profiling did not support a diagnosis of PGNT.^3^ By the DKFZ methylation classifier, the tumor in the current case was classified into reactive tumor microenvironment, possibly because the tumor purity in the submitted tissue was insufficient.

Glioneuronal tumors generally show indolent behavior with long progression-free survival.^3^ The clinical course of the present case with *GPRC5B*::*PRKCA* fusion has also been good, with a total of 9 years of follow-up without malignant transformation or massive expansion of the tumor. Although targeted treatments exist for other fusion-driven gliomas (eg, BRAF/MEK or TRK inhibitors), currently, no targeted therapies have been established for tumors with *PRKCA* fusions,^8^ and their clinical significance remains unclear. Current guidelines recommend molecular testing,^9^ and comprehensive molecular profiling, including DNA methylation analysis, may aid tumor classification and expand the understanding of the molecular landscape of rare glioneuronal tumors.

In summary, we report the first indolent low-grade glioneuronal tumor harboring a *GPRC5B*::*PRKCA* fusion, suggesting that novel fusion events may occur in tumors with atypical clinicopathological features.

## Supplementary Material

vdag156_Supplementary_Data

## Data Availability

Data are available from the corresponding author upon reasonable request.
